# Spatiotemporal dynamics of spermatogenesis: insights from high-resolution spatial transcriptomics and pseudotime trajectories in mouse testes

**DOI:** 10.3389/frph.2025.1747902

**Published:** 2026-01-12

**Authors:** Han Liang, Jianlong Sun, Zaoxu Xu, Defeng Fu, Hangyu Zhang, Xiaoran Zhou, Chen Li, Huihua Xia, Gailing Li, Renjie Liao, Qi Wang, Erkai Liu, Luyang Zhao, Yuanye Bao, Gufeng Wang

**Affiliations:** 1 Shenzhen Salus BioMed Co., Ltd., Shenzhen, Guangdong, China; 2 Shanghai Salus Life Sciences Co., Ltd., Shanghai, China

**Keywords:** mouse testes, pseudotime trajectories, spatial transcriptomics, spatiotemporal dynamics, spermatogenesis, subcellular resolution

## Abstract

The molecular basis of spermatogenesis, which is a tightly regulated spatiotemporal process in testicular seminiferous tubules where germ cell differentiation and somatic-germ cell interactions drive sperm production, remains incompletely understood. Histological staining techniques lack molecular resolution, while scRNA-seq loses spatial context. Conventional spatial transcriptomics (approx. 100 μm resolution) is too coarse-grained for testicular cells (10–20 μm in diameter), leading to mixed-cell signals. In this study, we used Salus-STS high-resolution spatial transcriptomics (∼1 μm resolution) and Salus Cellbins Algorithm to characterize the spatial transcriptomic profile of mouse testes at single-cell level. Integrating the spatial data with scRNA-seq via RCTD annotated major cell subtypes, whose distributions aligned with histology. Pseudotime and spatial gradient analyses revealed a basement membrane-to-lumen developmental axis, with luminal genes (e.g., *Prm2*) enriched in sperm maturation and basal genes (e.g., *mt-Nd4*) linked to mitochondrial metabolism—validated by PPI and GO analyses. The biological relevance of these marker genes is underscored by the fact that mutations in *Prm2* and *mt-Nd4* are known to be associated with human male infertility, highlighting their potential diagnostic value. This work enables high-resolution dissection of spermatogenesis’ spatiotemporal dynamics, providing insights into male reproductive biology.

## Introduction

Spermatogenesis is a tightly regulated spatiotemporal process that generates mature spermatozoa in the testes, relying on the sequential differentiation of germ cells and coordinated interactions with somatic cells. The testis exhibits a highly organized tissue architecture: seminiferous tubules, the functional units of spermatogenesis, contain germ cells arranged in a developmental gradient—from spermatogonia at the basement membrane to elongating spermatids near the lumen—while Leydig cells reside in the interstitial spaces between tubules (1). Decoding the molecular mechanisms governing this spatiotemporal order is critical for understanding male reproductive health, as disruptions in spermatogenesis are a major cause of human male infertility (2). However, traditional research approaches have been limited in capturing both molecular expression and spatial context, creating a gap in our understanding of this complex process.

Histological staining techniques provide insights into testicular morphology but lack the molecular resolution to link cellular structure to gene expression programs (3). Single-cell RNA sequencing (scRNA-seq) has advanced the identification of testicular cell types and their gene expression profiles, yet it dissociates cells from their native tissue environment, losing critical information about cell-cell interactions and spatial-dependent gene regulation (4). Conventional spatial transcriptomics technologies (e.g., Visium) address this limitation by preserving spatial coordinates of RNA molecules, but their relatively low resolution (100 μm spot spacing (5) exceeds the average size of testicular cells (10–20 μm in diameter (6). This results in RNA pooling from multiple cells within a single capture spot, precluding accurate cell segmentation, reliable cell type assignment, and the delineation of spatiotemporal gene expression patterns that drive spermatogenesis.

To address these challenges, high-resolution spatial transcriptomics tools enabling subcellular RNA capture and precise cell segmentation—integrating molecular expression with spatial architecture to decode cell identity-location-function interplay in the testis—are urgently required. In this study, we leveraged Salus-STS (Spatial Transcriptomic Solution; Salus BioMed Co., Ltd.), a novel spatial transcriptomic platform with 1 μm resolution (defined as the center-to-center distance between adjacent capture spots; the individual capture spot has a diameter of ∼0.8 μm), enabling subcellular characterization of individual testicular cells (7). Applied to mouse testicular tissue, we aimed to validate individual cell segmentation, annotate cell types via integration of spatial transcriptomics and scRNA-seq (with spatial distribution mapping), and identify/validate spatiotemporal markers linked to spermatogenesis' basement membrane-lumen gradient. This work successfully resolves individual testicular cells, accurately annotates major cell types (2 somatic and 4 germ cell subtypes), and uncovers functionally coherent spatiotemporal marker genes linked to key processes in spermatogenesis (e.g., sperm flagellum biogenesis, DNA condensation, and mitochondrial oxidative phosphorylation), thereby deepening our understanding of the molecular and spatial regulation of spermatogenesis and laying the groundwork for translational research in male infertility and reproductive biology.

## Results

### High-resolution spatial transcriptomics enables effective segmentation of mouse testicular cells at the subcellular level

**Figure 1:**
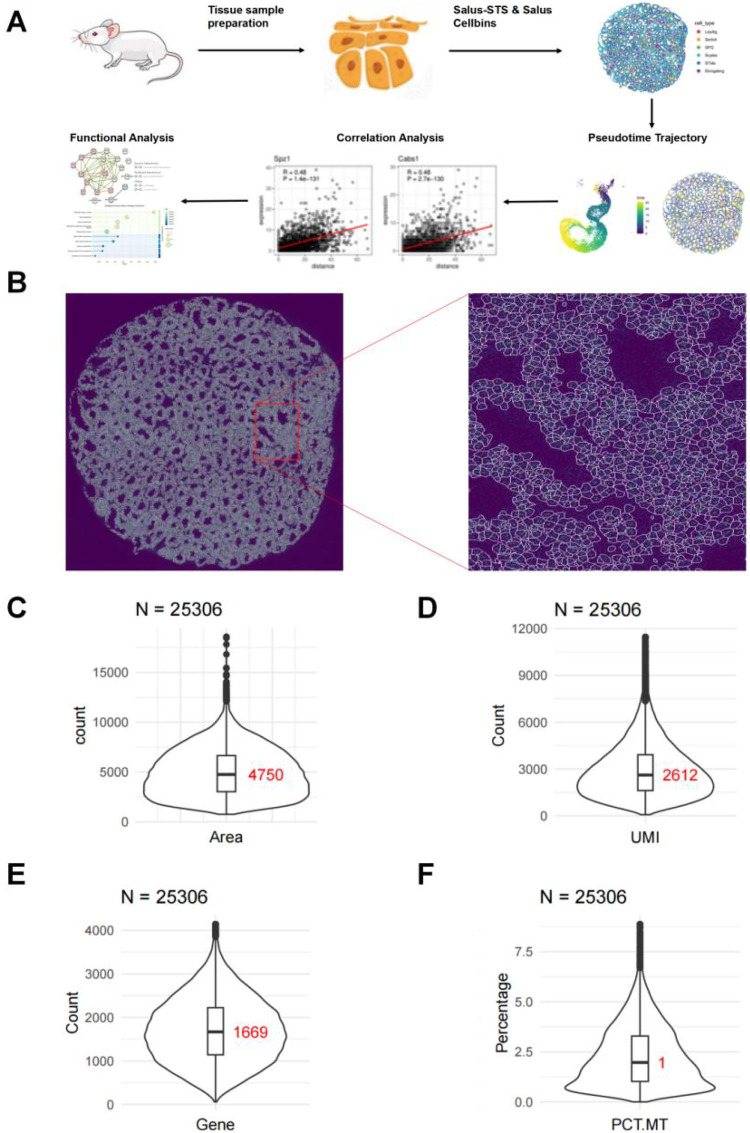
Salus-STS high-resolution spatial transcriptomics enables effective cell identification at the subcellular level. **(A)** Schematics illustrating of the study. **(B)** Results of cell segmentation via the Salus Cellbins Algorithm. **(C–F)** Distributions and medians (red text in the figures) of the area (in pixel^2^) **(C)**, UMI counts **(D)**, gene numbers **(E)**, and proportions of mitochondrial UMIs **(F)** of segmented cellbins.

Spatial transcriptomics preserves the spatial coordinate information of intracellular RNA through *in situ* capture. Conventional spatial transcriptomics technologies are constrained by inadequate resolution. For example, the spot spacing of Visium is 100 μm (5), which exceeds the average size of cells (typically 10–20 μm in diameter (6). This means that a single capture spot in Visium may contain RNA from multiple cells, precluding effective distinction of individual cells. In contrast, the spot spacing of Salus-STS is only 1 μm (7), thereby achieving subcellular resolution. Applying Salus-STS to mouse testes tissue, we constructed mouse testicular cell architectures, uncovered the functionally coherent spatiotemporal marker genes linked to key processes in spermatogenesis (Figure 1A).

High resolution spatial transcriptomics data generated by Salus-STS, together with an unspliced RNA-based cell segmentation algorithm (Salus Cellbins (7), enable an accurate segmentation of mouse testicular cells (Figure 1B). In total, 25,306 cells were successfully segmented from the mouse testicular tissue sample. Quantitative analysis of the segmented results showed that the median cross-sectional area of each cell was 4,750 pixel bins, equivalent to a circular area with a radius of approximately 39 pixels or a square with a side length of around 69 pixels (Figure 1C). Each pixel bin represents a physical area of 250 nm × 250 nm, resulting in the median cross-sectional cell area of approximately 300 μm^2^. This segmented cellular unit is referred to as a “cellbin”.

As illustrated in the right panel of Figure 1B, a subset of RNA molecules was localized outside the segmented cellular boundaries. Nevertheless, a high Unique Molecular Identifier (UMI) recall rate of 68% (74,697,589 out of 109,309,259) was still achieved. Among the segmented cells, the median number of RNA UMIs per cellbin was 2,612 (Figure 1D), with a median of 1,669 genes detected per cellbin (Figure 1E), a median mitochondrial UMI proportion of 1% (Figure 1F), and a saturation level of 0.78. These quantitative results validate the high molecule capture efficiency of the Salus-STS platform. Collectively, the high-resolution capture capability of Salus-STS and the precise segmentation performance of the Salus Cellbins Algorithm enable effective resolution of individual cells in complex testicular tissue, thereby laying a robust foundation for downstream spatiotemporal analyses of gene localization and functional investigations.

### Cellbin-based analysis bolsters accurate identification of testicular cell types

**Figure 2:**
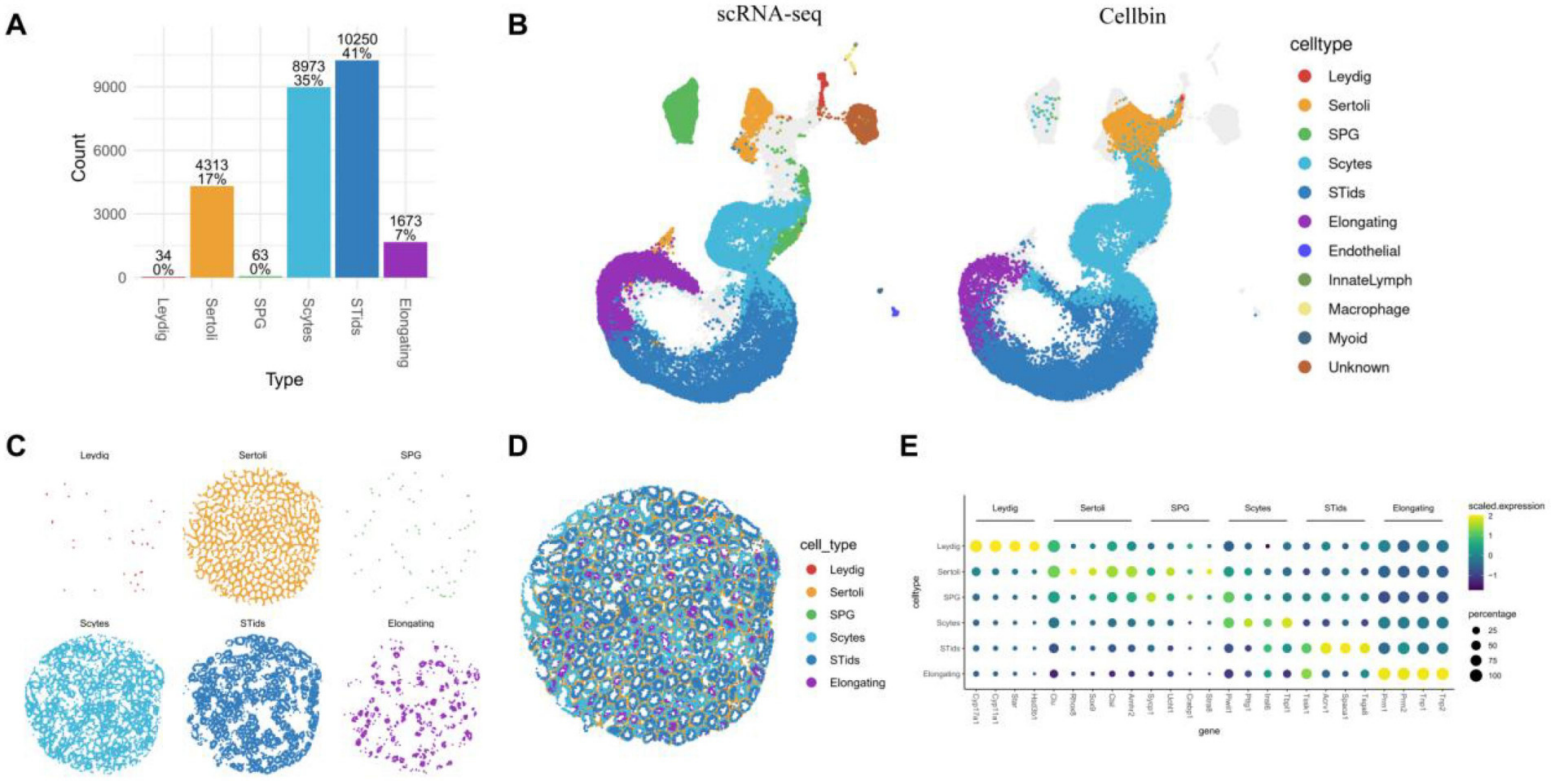
Cellbin-based analysis enables accurate identification of distinct cell types in the mouse testis. **(A)** RCTD-annotated distinct cell types and their proportions. **(B)** UMAP visualization of the Salus-STS Cellbin data with scRNA-Seq data. **(C)** Spatial distribution of distinct cell types in the mouse testis. **(D)** Integrated distribution map of cell distributions in the mouse testis. **(E)** Markers of distinct cell types and their expression levels. Scaled expression: the average expression level scaled across genes to eliminate the effect of total expression level differences among genes. Percentage: for each cell type, the percentage of cellbins that express the specific gene out of all cellbins of the same type.

We integrated Salus-STS-generated mouse testicular spatial transcriptomics data with mouse testicular scRNA-seq data from a previous study (GSE112393) (4), and then performed cell type annotation on segmented cellbins using the Robust Cell Type Decomposition (RCTD) algorithm. The 25,306 segmented cellbins were primarily identified as two types of supporting somatic cells (Leydig and Sertoli) and four types of germ cells (spermatogonia (SPG), meiotic spermatocytes (Scytes), postmeiotic haploid round spermatids (STids), and elongating spermatids (Elongating)) (Figure 2A). 107 cellbins (0.4%) could not be annotated and were excluded from subsequent analyses. To assess the consistency between Salus-STS data and scRNA-seq data, canonical correlation analysis (CCA) was used to integrate the two datasets, with Uniform Manifold Approximation and Projection (UMAP) employed for visualization. UMAP visualization demonstrated strong concordance in cell type distribution patterns between the two datasets, validating the authenticity of Salus-STS capture results (Figure 2B).

Furthermore, to dissect the topological arrangement of testicular tissue at high resolution, we systematically mapped the spatial distribution of all cell types across the entire tissue section (Figure 2C and Supplementary Figure S1), with an integrated distribution map provided in Figure 2D. These spatial maps not only illustrate the precise localization of each cell type within the testicular microenvironment but also reveal key organizational features: Leydig cells are enriched in the interstitial spaces between tubules, Sertoli cells are consistently positioned along the basement membrane of seminiferous tubules, and the four germ cell subtypes (SPG, Scytes, STids, and Elongating) are distributed within the seminiferous tubules.

The robustness of our cell type annotations was further validated by interrogating the expression patterns of well-characterized testicular marker genes across distinct cell clusters (Figure 2E). We selected marker genes with documented specificity in testicular biology, including: *Cyp17a1,* a key steroidogenic enzyme with 17α-hydroxylase and 17,20-lyase activity, critical for androgen biosynthesis in Leydig cells (8); *Clu* (clusterin), a secreted chaperone protein essential for Sertoli cell homeostasis and support of germ cell development (9); *Sycp1*, a component of the synaptonemal complex initiation complex involved in early germ cell differentiation (10); *Piwil1*, an Argonaute family protein required for meiotic progression and transposon silencing in spermatocytes (11); T*ssk1*, a testis-specific serine/threonine kinase involved in round spermatid maturation (12); and *Prm1*, a protamine responsible for chromatin condensation in elongating spermatids (13). The concordance between marker gene expression and cell type annotations provides strong evidence for the reliability of our classification.

### High-resolution spatial transcriptomics uncovers spatiotemporal markers of spermatogenesis

**Figure 3:**
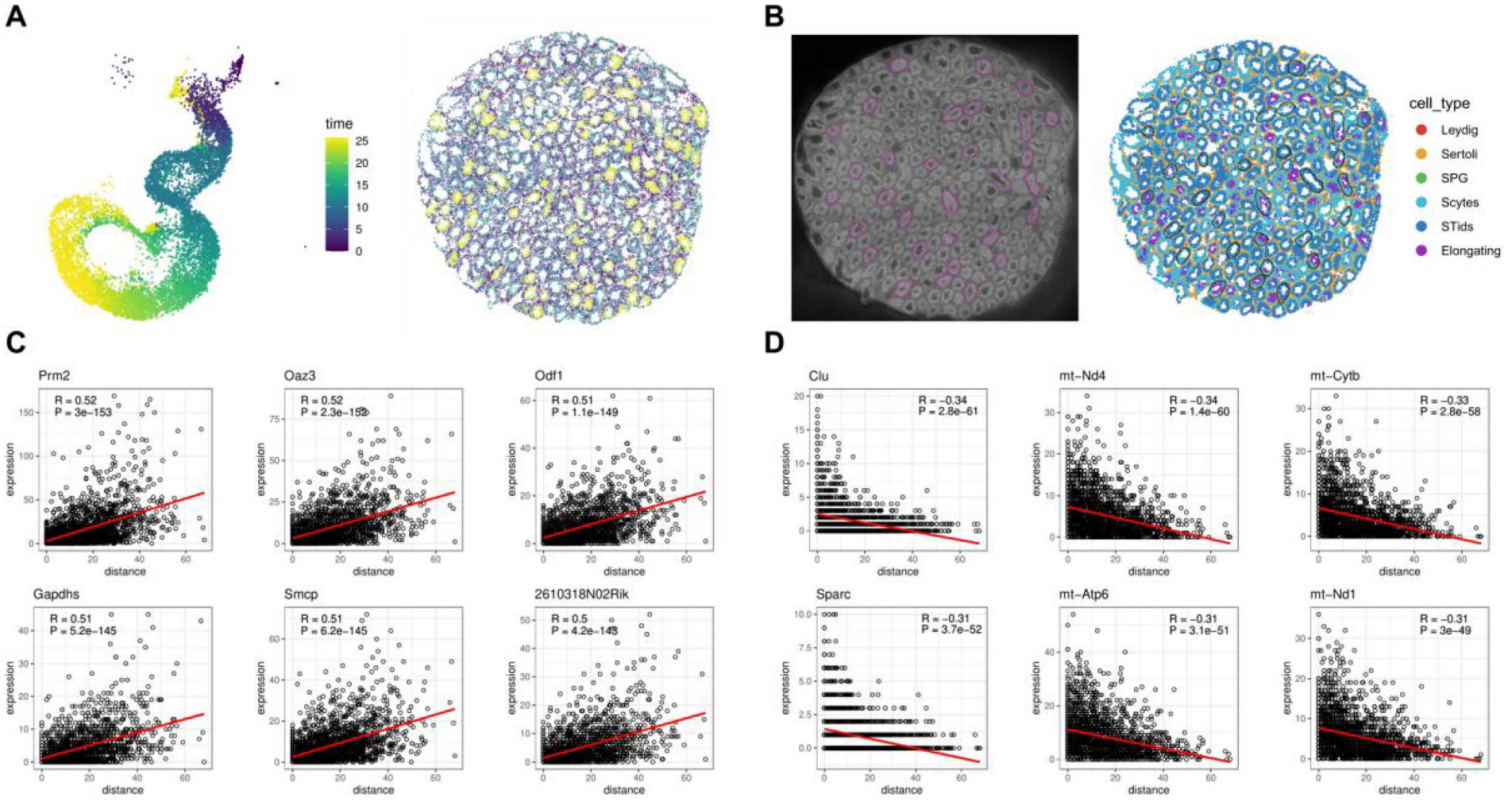
High-resolution spatial transcriptomics uncovers spatiotemporal markers of spermatogenesis. **(A)** Pseudotime trajectory analysis. **(B)** Randomly selected seminiferous tubules. **(C,D)** Top 6 genes with expression levels positively **(C)** and negatively **(D)** correlated with the axis from the tubule basement membrane (epithelium) to the lumen center respectively.

We performed pseudotime trajectory analysis and reconstructed the temporal sequence of spermatogenesis: progressing from SPG to Scytes, then to STids, and finally to Elongating cells (Figure 3A and Supplementary Figure S2). Concurrently, observations from the spatial map (Figure 2D) and pseudotime trajectory (Figure 3A) revealed that these four germ cell subtypes exhibit a spatiotemporal developmental gradient—Scytes localize peripherally, while Elongating cells concentrate toward the tubule lumen—consistent with the canonical spatiotemporal progression of spermatogenesis. Building on the spatial transcriptomics data from Salus-STS, which revealed consistency between the temporally resolved trajectory and the spatially observed developmental gradient, we sought to dissect the gene expression dynamics underlying this coordinated process. Specifically, we aimed to identify genes whose expression patterns track with the spatial axis of seminiferous tubules (from basement membrane to lumen), as such patterns likely reflect functional roles in driving stepwise germ cell differentiation.

To explore these spatiotemporal expression patterns, multiple seminiferous tubules were randomly and uniformly selected across the entire testicular section (Figure 3B), with cellular expression data from a total of 2,231 cellbins analyzed along the axis from the tubule basement membrane (epithelium) to the lumen center. This analysis revealed that the expression levels of some genes positively correlated with spatial distance from the basement membrane (i.e., expression increased toward the tubule lumen), including *Prm2*, *Oaz3*, *Odf1*, *Gapdhs*, *Smcp*, and *2610318N02Rik* (Figure 3C and Supplementary Figure S3A). Conversely, some genes showed an inverse trend (expression decreased toward the lumen), such as *Clu*, *mt-Nd4*, *mt-Cytb*, *Sparc*, *mt-Atp6*, and *mt-Nd1* (Figure 3D and Supplementary Figure S3B). The complete relationships between gene expression and spatial distance are listed in Supplementary Table S1.

### Functional validation of spatiotemporal markers in spermatogenesis

**Figure 4:**
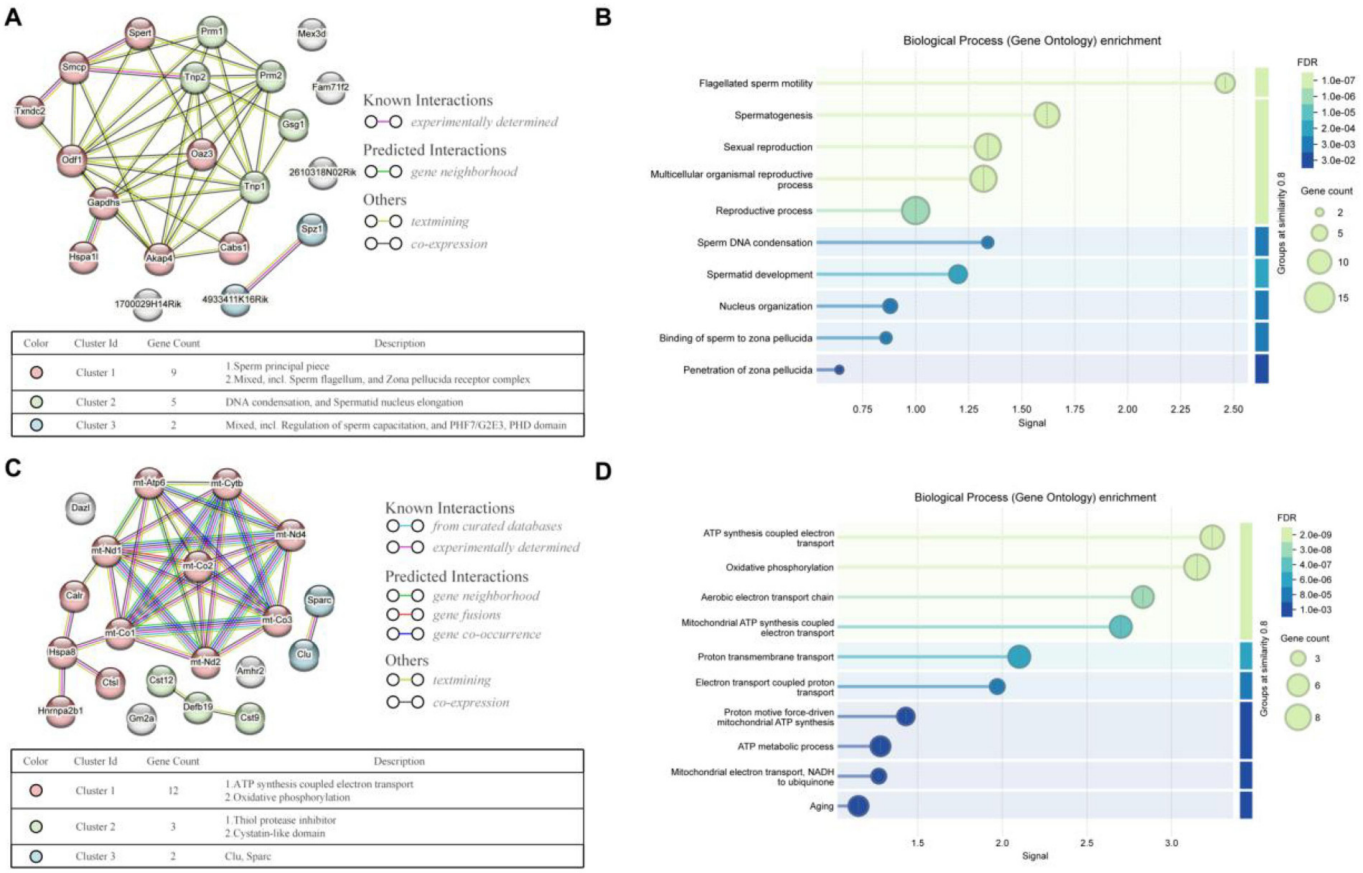
Functional analysis of spatiotemporal markers in spermatogenesis. **(A,B)** Protein-protein interaction (PPI) **(A)** and Gene Ontology (GO) Biological Process (BP) **(B)** analyses of the top 20 genes whose expression levels are positively correlated with the axis from the tubule basement membrane (epithelium) to the lumen center. **(C,D)** PPI **(C)** and GO BP **(D)** analyses of the top 20 genes with expression levels negatively correlated with the axis from the tubule basement membrane (epithelium) to the lumen center.

To validate the functional relevance of these spatiotemporal markers, we analyzed the top 20 genes with positive (Figure 3C and Supplementary Figure S3A) and negative (Figure 3D and Supplementary Figure S3B) correlations with distance from the basement membrane using protein-protein interaction (PPI) networks and Gene Ontology (GO) enrichment analyses. For positively correlated genes, the PPI network resolved three functional clusters: Cluster 1 (9 genes) underpinned sperm flagellum biogenesis and zona pellucida receptor complex formation, Cluster 2 (5 genes) regulated sperm DNA condensation and nuclear elongation, and Cluster 3 (2 genes) governed sperm capacitation (Figure 4A). GO Biological Process (BP) enrichment reinforced these roles, with top-ranked terms including “flagellated sperm motility”, “sperm DNA condensation”, and “spermatid development”, consistent with their enrichment near the tubule lumen (a niche of maturing spermatids) (Figure 4B). GO Cellular Component (CC) enrichment further localized their functions to sperm-specific structures (e.g., sperm flagellum and male germ cell nucleus), directly linking spatial distribution to structural maturation during spermatid differentiation (Supplementary Figure S4A).

In contrast, negatively correlated genes formed a PPI network centered on mitochondrial energy metabolism (Figure 4C), with GO BP enrichment prioritizing “ATP synthesis-coupled electron transport”, “mitochondrial ATP synthesis”, and “oxidative phosphorylation” (Figure 4D). GO CC enrichment mapped their activity to mitochondrial respiratory structures (e.g., respiratory chain complex and inner mitochondrial membrane), core components of oxidative phosphorylation (Supplementary Figure S4B). These metabolic functions align with the basal tubule region, where spermatocytes undergo energy-intensive meiotic divisions.

Collectively, these interactome and ontology analyses demonstrate that spatiotemporal marker genes are functionally coherent, integrating their spatial localization with stage-specific biological processes and subcellular architectures in spermatogenesis. This functional validation solidifies the role of these markers as molecular proxies for dissecting the spatiotemporal dynamics of sperm cell development.

## Discussion

This study systematically dissects the spatiotemporal dynamics of spermatogenesis in mouse testes using Salus-STS and reveals core mechanisms of cell fate determination and intercellular interactions during spermatogenesis, laying the groundwork for translational research in male infertility and reproductive biology.

Salus-STS features 1 μm resolution (defined as the capture spot center-to-center distance), which is well-suited to the size of individual testicular cells and enables subcellular RNA capture. Combined with Salus Cellbins Algorithm—which uses nuclear unspliced RNA as boundary markers and watershed segmentation—this approach segmented 25,306 cellbins (median cross-sectional area ∼300 μm^2^) with a median of 2,612 UMIs and 1,669 genes per cellbin, achieving a 68% UMI recall rate. Notably, we analyzed the distribution of recalled and unrecalled UMIs: among 28,407 detected genes, 19,443 (68.44%) genes were classified as significantly recalled (i.e., localized within cellbins), and 133 (0.47%) genes were classified as significantly unrecalled (Supplementary Table S2). The proteins encoded by these 133 genes were predominantly enriched in the extracellular space/region/matrix (Supplementary Figure S4C),—a result that partially validates the accuracy of our segmentation algorithm. This segmentation approach avoids the limitations of traditional pixel-wise segmentation, which mechanically divides regions into rigid square units in a rigid manner that fails to align with the actual morphological features of cells (14). Instead, it more accurately reflects the natural, irregular shapes of testicular cells. This synergy between the high-resolution RNA capture capability of Salus-STS and the morphology-adaptive segmentation capability of Salus Cellbins Algorithm solves the challenge of individual cell identification in dense testicular tissue, filling gaps in traditional spatial transcriptomics platforms.

Accurate cell annotation is critical for spatial transcriptomics, especially for germ cell subtypes with overlapping profiles. By integrating Salus-STS data with a published mouse testicular scRNA-seq dataset (GSE112393) (4) via the RCTD algorithm, we identified 2 somatic cell types (Leydig, Sertoli) and 4 germ cell subtypes (SPG, Scytes, STids, Elongating), with only 0.4% (107) unannotated cellbins excluded. CCA integration and UMAP visualization showed high consistency between spatial and scRNA-seq data, validating the reliability of the capture results. To further confirm the segmentation algorithm's generalizability, we performed alternative validation using a mouse brain tissue section subjected to both Salus Cellbins Algorithm and HE staining, showing high consistency between algorithm-delineated boundaries and HE-stained morphology (Supplementary Figure S5). Spatial localization aligned with histological features: Leydig cells in interstitial spaces (responsible for androgen synthesis (15), Sertoli cells along the basement membrane (responsible for germ cell support (16), and germ cells forming gradients in the tubules. Marker gene expression (e.g., *Cyp17a1* in Leydig cells, *Clu* in Sertoli cells, *Sycp1* in Scytes, *Prm1* in Elongating cells) further confirmed annotation accuracy. Notably, Sertoli-SPG colocalization supports the “spermatogonial stem cell niche hypothesis” (Sertoli cells secrete GDNF for SPG self-renewal (17), revealing intercellular functional interactions via spatial proximity.

Pseudotime trajectory analysis indicated that germ cells undergo developmental progression in a sequential manner, starting from spermatogonia, progressing to spermatocytes, then to spermatids, and finally to elongating spermatids. This developmental process exhibits a distinct spatial gradient, extending from the basement membrane of seminiferous tubules towards the lumen. Specifically, spermatocytes are located in the peripheral regions of the tubules, while elongating spermatids are positioned closer to the lumen. This phenomenon of “temporal-spatial consistency” prompted an in-depth analysis of 2,231 cellbins along the “basement membrane-lumen axis”. Through this analysis, two distinct gene groups were identified. One group consisted of genes that were upregulated toward the lumen, such as *Prm2*, *Oaz3*, and *Odf1*. The other group contained genes that were downregulated toward the lumen, including *Clu*, *mt-Nd4*, and *mt-Cytb*. We also performed targeted analysis of specific regulators, taking the transcription factor *Gata4* as an example: *Gata4* showed significant positive correlations with its reported target genes (*Pgam1* and *Pfkp*) (18) in both all cellbins and Sertoli cells alone, while other targets exhibited inconsistent correlations (Supplementary Figure S6). This inconsistency is attributed to “dropout”—a prevalent artifact in single-cell and spatial transcriptomics, driven by limited mRNA abundance and inefficient RNA capture in individual cells (19). To address this dropout-induced ambiguity and consolidate the reliability of regulatory markers, subsequent studies could combine computational imputation algorithms (19–22) and experimental validation (e.g., immunofluorescence staining, qPCR, and Western blot).

To further explore the functional coherence of these gene groups, PPI and GO analyses were conducted. The results of these analyses confirmed that the genes upregulated towards the lumen were significantly enriched in biological processes related to sperm flagellum biogenesis, sperm DNA condensation, and sperm capacitation. For instance, *Odf1*, a component of the outer dense fibers of spermatozoa, is crucial for maintaining the integrity of the sperm flagellum (23). *Prm2*, which substitutes for histones in sperm chromatin during spermatogenesis, is involved in sperm chromatin condensation (24). Conversely, the genes upregulated in the basal region of the tubules were predominantly enriched in processes related to mitochondrial oxidative phosphorylation. For example, *mt-Nd4*, a core subunit of the mitochondrial membrane respiratory chain NADH dehydrogenase (Complex I), participates in electron transfer from NADH to the respiratory chain; and *mt-Cytb* is a component of the ubiquinol-cytochrome c reductase complex (complex III), contributing to the generation of a proton gradient for ATP synthesis (25). This is in line with the high ATP demand of spermatocytes during meiosis (26). Additionally, *Sparc,* which is enriched in the basal region of the tubules, may contribute to maintaining the integrity of the basement membrane, thereby providing a suitable niche for spermatogonia (27). The biological significance of these markers is not arbitrary. Mutations in *Prm2 and mt-Nd4* have been associated with human male infertility (28–30), suggesting their potential as diagnostic markers.

However, this study is not without limitations. Firstly, the research focused primarily on normal adult mouse testes. As is well established, human testes possess distinct characteristics, such as longer seminiferous tubules and slower cell cycles (31). Therefore, it is essential to validate these markers in human tissue samples to ensure their clinical relevance. Secondly, the functional validation in this study relied primarily on bioinformatics approaches, specifically PPI and GO analyses. To establish causal relationships, *in vitro* experiments, such as RNA *in situ* hybridization, or *in vivo* experiments, such as CRISPR-mediated *Odf1* knockout, are necessary. Thirdly, the current analysis focuses exclusively on protein-coding genes to define spatiotemporal markers of spermatogenesis, while neglecting the role of non-coding RNAs (ncRNAs)—including microRNAs (miRNAs), long non-coding RNAs (lncRNAs), and PIWI-interacting RNAs (piRNAs)—that are critical for regulating spermatogenic processes. Future studies could reprocess Salus-STS to capture both protein-coding gene and ncRNA signals to construct a more comprehensive spatiotemporal regulatory network. Additionally, spatial transcriptomics alone cannot precisely localize fine subcellular structures such as cytoplasmic bridges (Supplementary Figure S7)—which are only 0.5–3 μm in diameter, connect germ cells at the same developmental stage, and enable intercellular substance exchange (32, 33). This is because the core of spatial transcriptomics relies on capturing RNA spatial distribution, which has limited correlation with the morphological delineation of such microscale structures; accurate localization of these structures instead requires analyzing the spatial patterns of proteins (e.g., membrane surface proteins, cytoskeletal proteins) that define their structural boundaries.

Several promising future research directions can be outlined. Firstly, studying human testicular tissue, including both normal and infertile samples, could help validate the markers and develop comprehensive diagnostic panels. Secondly, using the Salus-STS method in mouse infertility models, such as those induced by heat stress-induced arrest, may enable the identification of pathological pathways underlying male infertility. Thirdly, extending the application of Salus-STS to other reproductive tissues, such as mouse ovaries and human epididymis, could contribute to the exploration of conserved and tissue-specific regulatory mechanisms in the reproductive system. And to facilitate integrative multidisciplinary research, the application of Salus-STS can be extended to other related fields, including structural and systems biology, endocrine and reproductive physiology, molecular and reproductive endocrinology, and viral genomics and epigenomics. Additionally, multi-omics integration (e.g., with spatial proteomics) is needed for more precise reconstruction of cellular structures. We also plan to optimize the Salus-STS platform resolution, refine the segmentation algorithm, and develop an HE staining-based segmentation method to enhance cell boundary delineation accuracy.

In conclusion, by applying Salus-STS and Salus Cellbins Algorithm, we successfully dissected the spatial transcriptomic profiles of mouse testes at single-cell resolution, and uncovered functionally coherent marker genes linked to spermatogenesis' spatiotemporal dynamics. This work not only deepens our understanding of the molecular mechanisms underlying spermatogenesis but also lays a robust foundation for translating spatial omics insights into the field of male infertility research.

## Methods

### Mouse testis sample preparation

Animal experimental procedures in this study adhered to ethical norms for animal research and were approved by the Animal Care and Use Committee of the Guangzhou Institutes of Biomedicine and Health, Chinese Academy of Sciences (approval No. TOPGM-IACUC-2024-0255). A male C57BL/6J mouse (4 weeks old, purchased from Top Biotech, Shenzhen, China) was euthanized, and the testis was promptly dissected. Upon collection, the testis was embedded in optimal cutting temperature (OCT) compound, snap-frozen on dry ice, and stored at −80 ℃ until cryo-sectioning.

### Salus-STS library preparation

The Salus-STS library was prepared using Salus-STS gene expression kit (Shenzhen Salus BioMed Inc. Ltd., Shenzhen, Guangdong, China). The testis sample was microsectioned to 10-μm thickness using a Leica CM1950 cryostat and affixed to Salus-STS chips. Chips were immediately incubated at 37 ℃ on a slide dryer for 3 min, then immersed in pre-chilled methanol for fixation at −20 ℃ for 30 min. Testis sections were permeabilized with 0.1% pepsin in 0.1 M HCl buffer at 37 ℃ for varying durations (3, 6, 12, 18, 24 min), followed by washing in 0.01X SSC containing 5% RNase inhibitor. Reverse transcription was performed by incubating sections in RT solution (10 U/μL Maxima H Minus Reverse Transcriptase, 1 mM dNTPs, 5% RNase inhibitor in 1X Maxima RT buffer) at 42 ℃ for 3 h, then washing with 0.01X SSC supplemented with 5% RNase inhibitor. Sections were subsequently digested with Exonuclease I (2 U/μL in 1X Exo I buffer) at 37 ℃ for 1 h and washed with 0.01X SSC. Tissues were then digested with 80 mM KOH at room temperature for 15 min, neutralized with buffer EB (10 mM Tris-Cl, pH 8.5). To collect the cDNA library, cDNA was denatured with 80 mM KOH and neutralized with 1 M Tris-HCl (pH 7.0). The library was first amplified using 2.5 μM cDNA primers in Kapa Hifi HotStart ReadyMix under the following conditions: 95 ℃ for 3 min, 15 cycles of 95 ℃ for 30 s, 60 ℃ for 1 min, and 72 ℃ for 1 min, with a final extension at 72 ℃ for 2 min and hold at 4 ℃. The library with the highest cDNA yield across permeabilization timepoints was selected for subsequent steps. A 50-ng aliquot of cDNA was fragmented using in-house Tn5 transposase at 55 ℃ for 10 min, quenched with 0.02% SDS at room temperature for 5 min, and further amplified with 2 μM primers in Kapa Hifi HotStart ReadyMix. PCR conditions were: 95 ℃ for 5 min, 15 cycles of 95 ℃ for 20 s, 60 ℃ for 20 s, and 72 ℃ for 30 s, with a final extension at 72 ℃ for 5 min and hold at 4 ℃. DNA products were sequenced on a Salus Pro sequencer following the manufacturer's protocol, using paired-end (100 bp) mode.

### Salus-STS raw data processing

From Fastq files, SBCs were obtained from positions 1–30 of read 1, and UMIs from positions 88–97 of read 1. SBCs were aligned to pre-generated chip SBC data (including sequences and coordinates) from chip fabrication, allowing 1-bp mismatches to correct sequencing and PCR errors. Read 2 (carrying cDNA data) was mapped to the mouse reference genome (mm10) via STAR (34) and annotated to corresponding genes. UMIs distinguished distinct mRNA captures sharing the same SBC and removed PCR duplicates, enabling construction of a coordinate-linked expression profile. Mitochondrial and ribosomal RNA proportions were mapped and checked as additional quality controls.

### Tissue boundary identification

Tissue boundary recognition begins with automated segmentation of sequencing-based expression heatmaps using the Segment Anything Model (SAM v1.0, https://segment-anything.com/). For scenarios requiring manual boundary definition, a two-step protocol is applied: first, histological contours are manually annotated on expression heatmaps via ImageJ (Fiji distribution v1.54f); second, the Flood Fill algorithm in OpenCV (v4.7.0) is implemented with 8-neighborhood connectivity and morphological closing (kernel size = 5 × 5) to generate binary masks. Spatial barcodes within these masks are extracted through Otsu's threshold-based binarization.

### Salus cellbins algorithm

The watershed segmentation algorithm was utilized to identify cells from the RNA expression matrix. Specifically, concentrations of unspliced mRNA were used to demarcate cell cores, as unspliced RNA fragments are typically localized within cell nuclei—a property that aids significantly in recognizing cell boundaries and partitioning the chip into cell-specific regions. To achieve this, a Gaussian filter was initially applied to smooth the RNA count distribution (as described by the RNA expression matrix), thereby suppressing random noise. The watershed algorithm was then employed to detect signal clusters, which were designated as cell core regions. Subsequent to this, region boundaries were expanded to incorporate scattered signals between cores, with adjustments based on sample characteristics and project needs, while accounting for both regional size constraints and total RNA counts per cell.

### Cell annotation

Cell annotation was carried out by integrating single-cell RNA sequencing (scRNA-seq) data with spatial transcriptomic data generated by Salus-STS. RCTD (Reference-based Cell Type Deconvolution) was employed to resolve cell-type composition within each spatial spot. Single-cell data used for testis annotation was retrieved from https://www.ncbi.nlm.nih.gov/geo/query/acc.cgi?acc=GSE112393.

### Pseudotime analysis

Pseudotime analysis was performed using Monocle (v2.30.1) in the R environment (v4.3.3 or higher, for compatibility). Owing to constraints on available computational resources, only 20,000 randomly selected cellbins were included in the analysis. Prior to pseudotime inference, gene filtering was applied: genes expressed in fewer than 10 cellbins were excluded to reduce noise from low-abundance transcripts.

Differentially expressed genes (DEGs) along the pseudotime trajectory were identified using the differentialGeneTest() function built into Monocle. To focus on the most biologically relevant DEGs for downstream analyses (e.g., Unsupervised clustering), only the top 2,000 genes with the lowest Q-values (i.e., most statistically significant) were retained.

### Association analysis between gene expression and radial distance

To characterize the spatial variation of gene expression along the radial axis of seminiferous tubules (from the tubular membrane to the lumen center), we performed a correlation analysis between the gene expression level of each cellbin and its distance to the tubular membrane. For distance quantification, the boundaries of individual seminiferous tubules were manually outlined first; for each cellbin, Euclidean distances from its centroid to all pixels on the outlined tubular boundaries were calculated, and the minimum value among these distances was defined as the “distance of the cellbin to the tubular membrane”. For the expression-distance correlation analysis, the Pearson correlation coefficient was computed for each gene to assess the linear relationship between its expression levels in cellbins and the corresponding cellbin-to-membrane distances. Pearson correlation was calculated using the built-in cor.test() function in R version 4.4.0 with default parameters (alternative = “two.sided”, method = “pearson”, conf.level = 0.95).

### PPI and GO enrichment analysis

For protein-protein interaction (PPI) and Gene Ontology (GO) enrichment analyses, we selected three sets of genes: first, the top 20 genes whose expression exhibited positive correlation with the positional gradient along the axis from the tubule basement membrane (epithelium) to the lumen center; second, the top 20 genes with negative correlation to this gradient; and third, the 133 significantly unrecalled genes identified in the UMI distribution analysis. These gene sets were analyzed using The STRING database (Version 12; https://string-db.org/). Clustering was performed via the k-means algorithm, which partitions data into a predefined 3 clusters based on centroid-driven grouping.

## Data Availability

Salus-STS gene expression matrix reported in this paper have been deposited in the OMIX, China National Center for Bioinformation/Beijing Institute of Genomics, Chinese Academy of Sciences (https://ngdc.cncb.ac.cn/omix: accession no.OMIX011844). A standard workflow for converting fastq files into bin-segmented expression matrices has been publicly released, and the corresponding resource can be accessed via the GitHub repository: https://github.com/xuzaoxu/SalusSTS.

## Supplementary material

Supplementary Figure S1 Probability of each cellbin being assigned to a specific cell type. Score: Probabilistic weight of each cell type within a spatial transcriptomic spot, determined by the Robust Cell Type Decomposition (RCTD) algorithm. Supplementary Figure S2 Pseudotime trajectory analysis. **(A)** Unsupervised clustering of marker genes from pseudotime trajectory analysis. **(B)** Temporal distribution of four germ cell types. Weight: We divided the pseudotime into 20 windows and then calculated the average weight of four germ cell types. The weight came from the RCTD score of each cell type, and it reflected the signal strength (probability) of the cell types. Supplementary Figure S3 Spatiotemporal markers in spermatogenesis (continued). **(A,B)** Genes ranked 7–20 with expression levels positively **(A)** and negatively **(B)** correlated with the axis from the tubule basement membrane (epithelium) to the lumen center, respectively. Supplementary Figure S4 Gene Ontology (GO) cellular component (CC) analyses. **(A,B)** The top 20 genes whose expression levels are positively **(A)** and negatively **(B)** correlated with the axis from the tubule basement membrane (epithelium) to the lumen center, respectively. **(C)** 133 genes with significant non-recallable UMI counts. Supplementary Figure S5 Validation of Salus Cellbins Algorithm via HE staining in mouse brain tissue. Supplementary Figure S6 Expression correlation analysis of transcription factor *Gata4* and its target genes. **(A)** Across all cellbins. **(B)** In Sertoli cellbins. *X*-axis: *Gata4* UMI count per cellbin; *Y*-axis: UMI count of target genes per cellbin; Circle size represents the number of cellbins. Supplementary Figure S7 Spatial distribution of cytoplasmic bridges-related genes (*Tex14*, *AKAP4*, *Prm2*) in mouse testicular germ cells.

Supplementary Table S1 Correlation of expression levels of all mouse genes across 2,231 cellbins with the axis from the tubule basement membrane (epithelium) to the lumen center. The *R* value (correlation) and *p* value were provided by the built-in function cor.test of R (Version 4.3.3).

Supplementary Table S2 Analysis of Recalled and Unrecalled UMIs. This table presents the results from a differential analysis comparing recalled (in cellbins) and unrecalled (out cellbins) UMI counts for each gene. Listed for every gene are the raw counts, proportional distribution, *p*-value (derived from an exact binomial test under the null hypothesis of equal distribution), False Discovery Rate (FDR)-adjusted *q*-value, and the final significance call.
